# Trabectedin plus pegylated liposomal doxorubicin (PLD) for patients with platinum-sensitive recurrent ovarian cancer: a prospective, observational, multicenter study

**DOI:** 10.1007/s00432-018-2637-1

**Published:** 2018-04-06

**Authors:** Ingo B. Runnebaum, Dietmar Reichert, Uta Ringsdorf, Markus Kuther, Tobias Hesse, Jalid Sehouli, Pauline Wimberger

**Affiliations:** 1 Department of Gynecology and Reproductive Medicine and ESGO Training Center for Gynecologic Oncology, Jena University Hospital, University Women’s Hospital, Am Klinikum 1, 07747 Jena, Germany; 2 Medizinische Studiengesellschaft NORD-WEST GmbH, Medical oncology, Kuhlenstraße 53d, 26655 Westerstede, Germany; 3 Lahn-Dill-Kliniken GmbH, Gynecology and Obstetrics, Forsthausstraße 1, 35578 Wetzlar, Germany; 4 Municipal Hospital Kiel, Medical oncology, Chemnitzstraße 33, 24116 Kiel, Germany; 5 Department of Gynecology and Obstetrics, Agaplesion Diakonieklinikum Rotenburg GmbH, Elise-Averdieck-Strasse 17, 27356 Rotenburg (Wümme), Germany; 6 Department of Gynecology with Center for Oncological Surgery, Campus Virchow Klinikum, Benjamin Franklin Charité Comprehensive Cancer Center and European Competence Center for Ovarian Cancer, Medical University of Berlin, Augustenburger Platz 1, 13353 Berlin, Germany; 7 Medical Faculty and University Hospital Carl Gustav Carus, Gynecology and Obstetrics, TU Dresden, Fetscherstr. 74, 01397 Dresden, Germany

**Keywords:** Trabectedin, Observational trial, Pegylated liposomal doxorubicin (PLD)

## Abstract

**Purpose:**

The OVA-YOND study is the first prospective, non-interventional trial designed to evaluate trabectedin (1.1 mg/m^2^) plus PLD (30 mg/m^2^) in patients with platinum-sensitive recurrent ovarian cancer (ROC), given according to the marketing authorization in real-life clinical practice across Germany.

**Methods:**

Eligible patients were adults with platinum-sensitive ROC, pretreated with ≥ 1 platinum-containing regimen/s. The primary endpoint was to assess safety/tolerability of the combination.

**Results:**

Seventy-seven patients with platinum-sensitive relapse from 31 sites were evaluated. Patients received a median of 6 cycles (range 1–21) with 39 patients (50.6%) receiving ≥ 6 cycles. Median treatment duration was 4.2 months (range 0.7–18.8), mostly on an outpatient basis (88.3% of patients). Most common grade 3/4 trabectedin-related adverse events (AEs) were leukopenia (18.2%), neutropenia (15.6%), thrombocytopenia (9.1%), alanine (7.8%) and aspartate aminotransferase (6.5%) increase, and nausea/vomiting (5.2% each). Neutropenia (18.2%), leukopenia (15.6%), thrombocytopenia (10.4%), and nausea/vomiting (5.2% each) were the most frequent grade 3/4 PLD-related AEs. No deaths attributed to drug-related AEs or unexpected AEs occurred. Five patients (6.5%) had a complete response and 19 patients (24.7%) achieved a partial response for an objective response rate of 31.2% with median response duration of 6.25 months. Sixteen patients (20.8%) had disease stabilization for a disease control rate of 51.9%. Median progression-free survival was 6.3 months and median overall survival was 16.4 months.

**Conclusion:**

Trabectedin plus PLD confer clinically meaningful benefit to pre-treated patients with platinum-sensitive ROC, being comparable to those previously observed in selected populations from clinical trials and with a manageable safety profile.

## Introduction

Ovarian cancer is an acronym for a group of different cancers that are derived from different, often non-ovarian tissues, resulting in the different cancer histotypes with diverse molecular heterogeneity, genetic instability and mutagenicity (Bast et al. 2009; The Cancer Genome Atlas Research Network 2011; Vaughan et al. 2011). Ovarian carcinoma is one of the most common gynecologic malignancies and the fifth most frequent cause of death by cancer in women with around 125,000 deaths annually worldwide (Torre et al. 2015). Roughly 75% of women with ovarian cancer present advanced stage of disease associated with poor outcome, and 50% of all cases occur in women over 65 years of age. Despite of high response rates (up to 80%) to the standard front-line treatment for advanced disease consisting of cytoreductive surgical debulking followed by platinum/taxane-based chemotherapy, after several platinum-based chemotherapy cycles approximately 75% of patients eventually relapse developing incurable drug-resistant, particularly platinum-resistant, disease with an overall 5-year survival rate lower than 50% (Cannistra 2010; Colombo et al. 2006; Corrado et al. 2017; Harter et al. 2010). As a result, identification of new treatment options for recurrent ovarian cancer (ROC), such as platinum-free regimens, represents an utmost clinical challenge.

Trabectedin (Yondelis^®^; PharmaMar, S.A., Madrid, Spain) is a natural drug derived from the marine tunicate *Ecteinascidia turbinata* and currently produced synthetically (ATC code: L01CX01). Trabectedin has pleiotropic mechanisms of action as in addition to act as a DNA-binding agent, inducing direct growth inhibition and ultimately apoptosis, it also has also selective anti-inflammatory and immunomodulatory properties, which promote tumor growth, angiogenesis and metastasis (D’Incalci 2013; D’Incalci and Galmarini 2010; Larsen et al. 2016). Following the initial approval of trabectedin in 2007, being the first ever marine-derived antineoplastic drug approved (Demetri et al. 2009), based on the results from a phase III randomized OVA-301 study (ClinicalTrials.gov Identifier: NCT00113607), which compared pegylated liposomal doxorubicin (PLD) alone with the combination of PLD and trabectedin in patients with ROC, in 2009 trabectedin plus PLD obtained the second marketing authorization for the treatment of patients with platinum-sensitive ROC (Monk et al. 2010). According with the recent survey carried out in Italy about the real-world management of trabectedin plus PLD in patients with platinum-sensitive ROC, currently, among the non-platinum/non-taxane treatments, this combination is the most frequently adopted regimen in such patients (Ferrandina et al. 2017). Particularly for patients suffering from platinum-induced toxicities or hypersensitivity, patients who had received more than one platinum-based chemotherapy or patients with partially platinum-sensitive disease who can benefit from a delay in platinum re-treatment (Poveda et al. 2014) the combination of trabectedin plus PLD represents an alternative in treating patients with platinum-sensitive relapse.

Thus far no prospective, non-interventional study with trabectedin plus PLD had been performed in a routine clinical setting, with a more diverse patient population with platinum-sensitive ROC than that recruited in clinical trials. Such an observational study can provide useful insights of the real-world toxicity, efficacy and management of patients receiving trabectedin plus PLD. Noteworthy, OVA-YOND study enrolled patients who may be underrepresented in clinical trials as it also considered patients with multiple comorbidities. Therefore, the prospective, non-interventional OVA-YOND trial (ClinicalTrials.gov Identifier: NCT01869400) was designed to evaluate the use of trabectedin plus PLD in patients with platinum-sensitive ROC and its safety and efficacy in routine clinical practice across Germany.

## Methods

### Study design

This prospective, multicenter, observational study evaluated trabectedin plus PLD in routine clinical practice to assess the toxicity and efficacy of the combination in women with platinum-sensitive ROC when given in accordance with the marketing authorization. Consistent with the real-life observational nature of the study, the normal clinical treatment routine was not altered for the patients included in the study as no additional per protocol diagnostic or therapeutic measures were required during the study. The primary endpoint of this study was to assess safety and tolerability of this non-platinum combination. Secondary endpoints included objective response rate (ORR), measured by Response Evaluation Criteria in Solid Tumors (RECIST) v.1.0 or v.1.1 (Eisenhauer et al. 2009; Therasse et al. 2000) and/or according to serum concentrations of cancer antigen (CA)-125. Secondary endpoints also comprised the assessment of the disease control rate (DCR), defined as the percentage of patients with a complete response (CR) or partial response (PR) and/or stable disease (SD), treatment and response duration, time-to-event variables [progression-free survival (PFS), time to progression (TTP) and overall survival (OS)], time to next treatment and the post-marketing data collection.

All study procedures were conducted in accordance with the ethical standards as laid down in the 1964 Declaration of Helsinki and its later amendments, guidelines for Good Pharmacoepidemiology Practice and the German Drug Law (AMG, § 67[6]), and were approved by the institutional review boards of each participating center. Signed informed consents were obtained from all study participants included in the study.

### Patients and treatments

Eligible patients were adults (> 18 years old) with advanced and histologically proven ovarian cancer, pretreated with one or more platinum-containing regimens and experiencing recurrence after a platinum-free interval (PFI) of ≥ 6 months, indicating a platinum-sensitive disease (Friedlander et al. 2011). Other eligibility criteria included adequate baseline renal, hepatic and bone marrow function according to laboratory standard parameters according to the Summary of Product Characteristics (SPC) 14 days before the first treatment cycle, complete recovery from any toxicity derived from prior treatment/s and no contraindication to dexamethasone or similar drugs. In accordance with the SPC excluded were patients who experienced disease progression during the last line of platinum-based chemotherapy or within 4 weeks of last platinum dose (i.e., with platinum-refractory disease), and those with a PFI < 6 months (i.e., with platinum-resistant disease), patients who received cumulative dose of doxorubicin > 400 mg/m^2^ or epirubicin > 720 mg/m^2^, and patients with prior history of clinically significant heart disease, such as angina pectoris, myocardial infarction within the last 6 months, severe ventricular arrhythmia, or acute ischemic disease. Women of childbearing age not using adequate contraception, pregnant and breastfeeding were also ineligible.

Trabectedin and PLD were administered in accordance with the SPC and the treating clinician’s discretion depending on the patient’s conditions and previous chemotherapy. The recommended dose of the combination for the treatment of platinum-sensitive ROC is PLD 30 mg/m^2^ immediately followed by trabectedin 1.1 mg/m^2^, administered as an intravenous infusion over 3 h every 3 weeks. Pretreatment with corticosteroids (e.g., dexamethasone 20 mg intravenously 30 min before PLD) was considered mandatory for all patients receiving trabectedin. If needed, additional anti-emetics could be administered in accordance with local practice. There were no pre-defined limits to the number of administered cycles and the treatment could continue until progressive disease, severe toxicity, consent withdrawal, or patient death. Following the treatment period, the follow-up period continued for 1 year after the last on-study administration with follow-up visits after approximately 6 and 12 months. Once trabectedin plus PLD treatment was discontinued, patients could have been treated with subsequent anticancer therapies or supportive care as per the clinician’s clinical judgment.

### Study evaluations

Adverse events (AEs) and serious adverse events (SAEs) were documented starting from the first application of trabectedin plus PLD and repeated at the treating clinician’s typical schedule until 30 days after administration of the last dose. After this period only AEs and SAEs related to trabectedin or PLD were collected. Tumor response was assessed according to the treating clinician’s usual clinical practice at baseline, during each treatment visit, and at follow-up visits at around 6 and 12 months after study’s end. According with the observational nature of this trial, responses measured either by RECIST or CA-125 levels expressed the best unconfirmed response obtained in any evaluation. Regardless of the used method no further confirmatory or re-staging response evaluations were required.

### Statistical methods

All statistical analyses had an exploratory nature and were presented in a descriptive manner with no aim to confirm or reject pre-defined hypotheses. The safety and efficacy analyses were based on all-treated population, defined as all patients who received at least one dose of trabectedin plus PLD. All AEs were coded using the Medical Dictionary for Regulatory Activities (MedDRA), v.15.0 and graded according to the National Cancer Institute-Common Terminology Criteria (NCI-CTC), v. 4.0. Time-to-event endpoints (PFS, TTP, and OS) and their fixed-time estimations were estimated according to the Kaplan–Meier method and were compared using the log-rank test. All *p* values were descriptive in nature and the significance level selected was 0.05. The TTP, PFS and OS analyses were defined as the time interval from the first administration of trabectedin plus PLD to the earliest date of disease progression for TTP, or until the earliest date of disease progression or death, regardless of cause (whichever occurred first) for PFS, whereas OS was defined as the time between the start of trabectedin plus PLD and patient death from any cause. Patients without tumor progression or death at the time of the final analysis or considered lost to follow-up were censored at their last date of radiological tumor assessment.

## Results

### Patient disposition and characteristics

From 18 April 2013 to 11 February 2016, a total of 77 out of 83 enrolled patients from 31 sites across Germany received trabectedin plus PLD and were included in the analysis set. All patients had platinum-sensitive ROC with a median PFI of 12 months (range 6–86 months) following the front-line platinum-based chemotherapy (Table 1). Patients had a median age of 66 years (range 40–78) and a good Eastern Cooperative Oncology Group (ECOG) performance status score of 0/1 was recorded in 62 patients (80.5%). At initial diagnosis, serous carcinoma was the most prevalent histological type (*n* = 54; 70.1%), being localized at the ovary in 68 patients (88.3%). All patients had metastatic disease, mostly located in peritoneum (35.1%) or liver (23.4%). Initially, all had undergone cytoreductive surgical debulking, which resulted in compete resection in 20 patients (26%), optimal debulking (visible residual tumor of ≤ 1 cm) was achieved in 11 patients (14.3%), whereas 16 patients (20.8%) had only palliative surgery as residual tumoral implants were > 1 cm. Noteworthy, 18 patients (23.4%) received secondary cytoreductive surgery, with complete resection in four and optimal debulking in two patients. Most patients were exposed to one or two lines of prior chemotherapy (*n* = 59; 76.6%) with a median of two lines of chemotherapy (range 1–6) prior to trabectedin plus PLD administration. Overall, > 60% of patients achieved a complete or partial response to the last chemotherapy treatment (Table 1).

**Table 1:** Patient and disease characteristics at baseline

	Evaluable patients (*n* = 77) *n* (%)	
Age at study entry (years)	Median (range)	66.0 (40.0–78.0)
Tumor location at initial diagnosis	Ovary	68 (88.3)
	Tubes	5 (6.5)
	Peritoneum	4 (5.2)
Histopathology	Serous	54 (70.1)
	Endometrioid	6 (7.8)
	Other/unknown	17 (22.1)
Federation of Gynecology and Obstetrics (FIGO) stage at diagnosis	IC	2 (2.6)
	IIIA	3 (3.9)
	IIIB	8 (10.4)
	IIIC	38 (49.4)
	IV	15 (19.5)
	Unknown	11 (14.3)
Platinum-free interval (months)	Median (range)	12 (6–86)
Eastern Cooperative Oncology Group (ECOG) performance status	0	29 (37.7)
	1	33 (42.9)
	0–1	62 (80.5)
	2	2 (2.6)
	Not available	13 (16.9)
Prior treatments	Prior surgery	77 (100)
	Complete resection	20 (26.0)
	Residual tumor ≤ 1 cm	11 (14.3)
	Residual tumor > 1 cm	16 (20.8)
	Unknown residual tumor	30 (39.0)
	Prior secondary surgery	18 (23.4)
	Complete resection	4 (5.2)
	Residual tumor ≤ 1 cm	2 (2.6)
	Residual tumor > 1 cm	3 (3.9)
	Unknown residual tumor	9 (11.7)
	Prior radiotherapy	3 (3.9)
	Prior chemotherapy	77 (100)
No. of chemotherapy lines prior to trabectedin plus pegylated liposomal doxorubicin (PLD)	Median (range)	2.0 (1–6)
	One prior line	26 (33.8)
	Two prior lines	33 (42.9)
	Three prior lines	12 (15.6)
	Four prior lines	5 (6.4)
	Six prior lines	1 (1.3)
Response to last chemotherapy	Complete response (CR)	23 (29.9)
	Partial response (PR)	25 (32.5)
	Stable disease (SD)	16 (20.8)
	Progressive disease (PD)	8 (10.4)
	Non evaluated (NE)	5 (6.5)

### Extent of exposure

Patients received a median of 6 trabectedin plus PLD cycles, with 39 (50.6%) patients receiving ≥ 6 cycles and up to a maximum of 21 cycles (Table 2). Patients received a median trabectedin/PLD dose intensity of 0.3/7.9 mg/m^2^/week and a respective median cumulative dose of 5.4/141.7 mg/m^2^, over a median treatment duration of 4.2 months (range 0.7–18.6), which represented 81.4%/79.3% of the planned dose intensity. The vast majority of patients were treated on an outpatient basis (*n* = 68, 88.3%) and received a total of 376 of 410 cycles (91.7%).

**Table 2:** Trabectedin plus PLD exposure

Trabectedin plus PLD treatment delivery	Evaluable patients (*n* = 77); *n* (%)	
Time on treatment (months)	Median (range)	4.2 (0.7–18.6)
Cycles per patient from the study enrollment	Median (range)	6.0 (1–21)
	1 cycle	8 (10.4)
	2 cycles	14 (18.2)
	3 cycles	5 (6.5)
	4 cycles	6 (7.8)
	5 cycles	5 (6.5)
	6 cycles	21 (27.3)
	≥ 7 cycles	18 (23.4)
Cycle duration (days)^a^	Median (range)	21.0 (15.0–95.0)
Cumulative dose (mg/m^2^)^b^	Median (range); trabectedin/PLD	5.4 (0.83–23.3)/141.7 (23.0–627.0)
Dose intensity (mg/m^2^/week)	Median (range); trabectedin/PLD	0.3 (0.2–0.5)/7.9 (2.9–10.2)
Relative dose intensity (%)	Median (range); trabectedin/PLD	81.4 (53.5–125.2)/79.3 (29.3–101.7)
Types of treatment	Outpatients	68 (88.3)
	Inpatients	2 (2.6)
	Both	7 (9.1)

Data shown are numbers and percentage of patients or median and range values with available data

^a^Duration of last cycle was set to 3 weeks. In all other cases, time between administrations was used

^b^Administered dose divided by body surface area (BSA)

Cycle delays occurred in 178 of 410 cycles (43%), commonly due to organizational/scheduling (63 cycles, 15.4%) reasons followed by hematological (55 cycles, 13.4%) or non-hematological toxicity (26 cycles, 6.3%). Similarly, dose reductions of trabectedin and PLD were frequent as occurred in 55% of cycles. Rounding a given calculated dose (trabectedin: 31.2%/PLD: 38.0% of cycles) and toxicity (trabectedin and PLD: ~ 10% of cycles each) were the most common reasons leading to dose reductions. The most common cause for therapy discontinuation was disease progression (*n* = 21, 27.3%), followed by patient’s wish (*n* = 11, 14.3%), non-hematological and hematological toxicity in 12 (15.6%) and six (7.8%) patients, respectively, tumor-related death (*n* = 9, 11.7%) and other reasons (*n* = 18, 23.4%).

### Efficacy

**Fig. 1:**
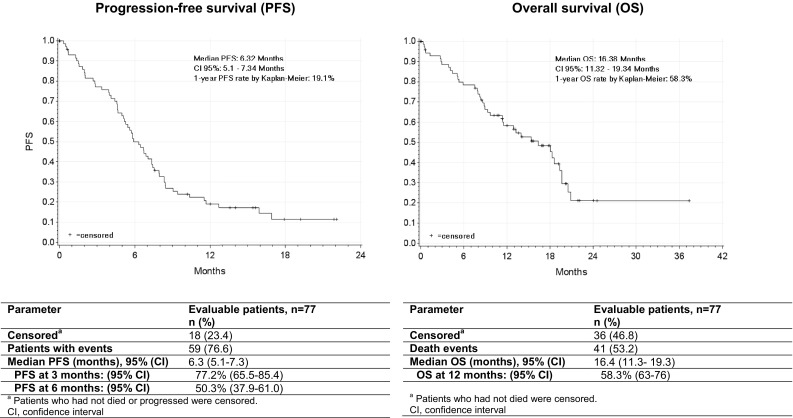
Kaplan–Meier plots of progression-free survival and overall survival

Regardless of the method of evaluation, five patients (6.5%) obtained a complete response and 19 patients (24.7%) achieved a partial response, reaching the ORR of 31.2% with the median duration of response of 6.25 months (Table 3). Additionally, 16 patients (20.8%) had disease stabilization as a best response for a DCR of 52.0%. Neither univariate nor multivariate regression analysis regarding the influence of parameters such as AEs (i.e., weight loss/gain, ascites/pleural effusion, abdominal pain) or concomitant medication on ORR found any statistically significant result. Analysis of PFS data was performed following a total of 59 progression or death events recorded (76.6% of patients), whereas 18 patients (23.4%) who were alive or were not assessed for disease progression at the time of this analysis were censored. Median PFS was 6.3 months [95% confidence interval (CI): 5.1–7.3], with 77.2 and 50.3% of patients free from progression at 3 and 6 months after treatment, respectively (Fig. 1). Among the patients who presented disease progression a median TTP of 7.3 months was recorded (95% CI 5.8–8.5). Furthermore, after 41 death events (53.2% of patients) treatment with trabectedin plus PLD resulted in a median OS of 16.4 months (95% CI 11.3–19.3), with 58.3% of patients alive 12 months after treatment (Fig. 1).

**Table 3:** Response assessment of trabectedin plus PLD

Best response (unconfirmed)	Evaluable patients (*n* = 77) *n* (%)
Complete response (CR)	5 (6.5)
Partial response (PR)	19 (24.7)
Stable disease (SD)	16 (20.8)
Progressive disease (PD)	9 (11.7)
Not evaluable	1 (1.3)
Missing	27 (35.1)
Objective response rate (ORR; CR + PR) [95% confidence interval]	14 (31.2) [21.1–42.7]
Disease control rate (DCR; ORR + SD) [95% confidence interval]	40 (52.0) [40.3–63.5]
Duration of response (DOR)^a^; months [95% confidence interval]	6.25 [3.36–9.44]

^a^Assessed on 24 patients

At the therapy end, the number of patients with ECOG performance status score of 0/1 decreased to 50.6% (*n* = 39). On the other hand, 39 patients (50.6%) either gained (*n* = 10, 13%) or did not change (*n* = 29, 37.7%) their bodyweight compared to 38 patients (49.4%) who experienced a decrease in their bodyweight between baseline and the end of therapy. No significant changes were observed regarding vital signs (i.e., heart rate, blood pressure and electrocardiogram) throughout the study.

### Safety

**Fig. 2:**
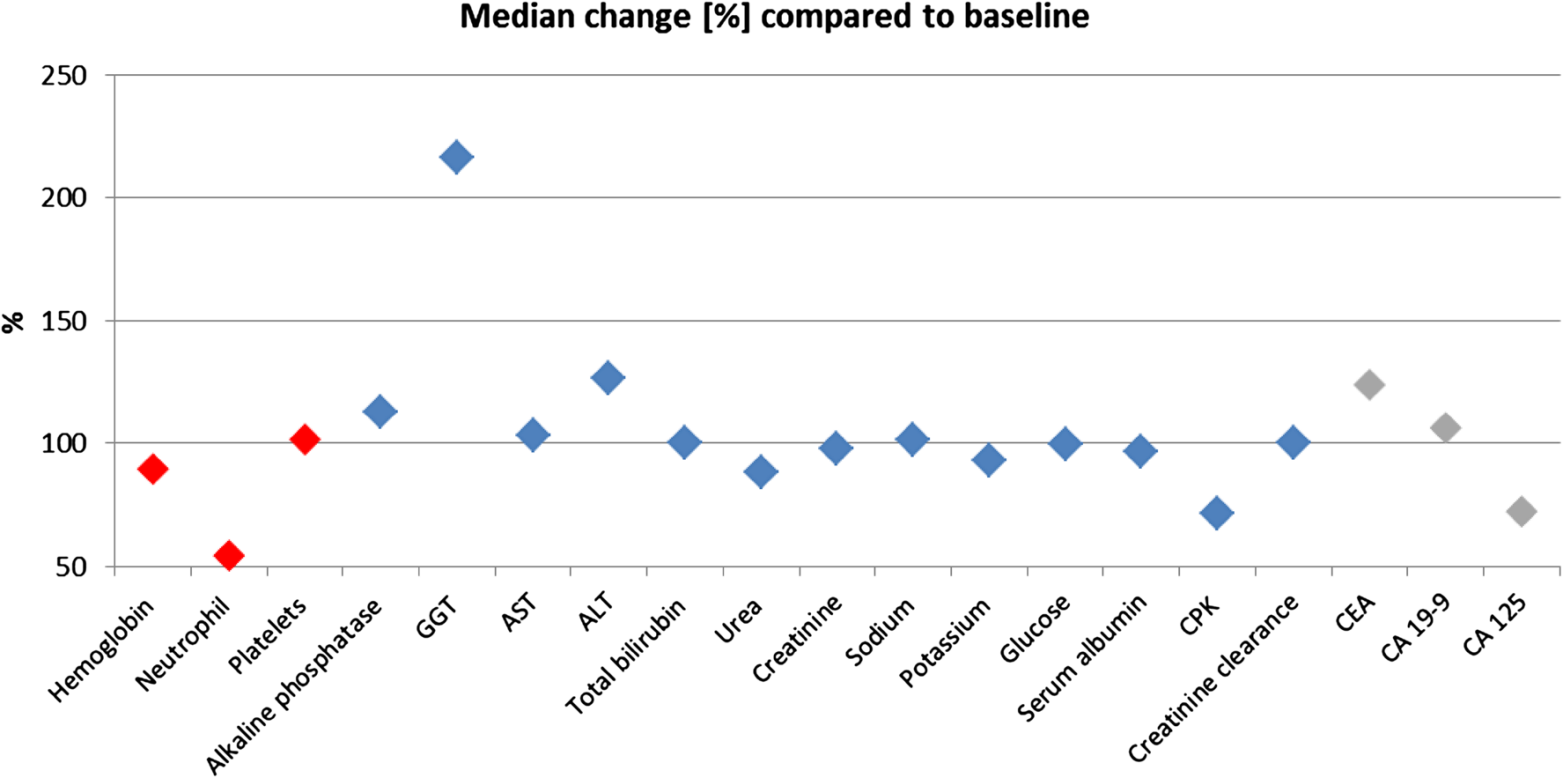
Changes in clinical laboratory parameters of the last individual assessment compared to baseline. Change of hematological (in red), biochemical (in blue) and tumor marker (in gray) as compared to baseline values is shown by setting the median baseline values as 100% and giving the percentages of change of the median values taken from the last individual assessment. ALT, alanine aminotransferase; AST aspartate aminotransferase; CA-125, cancer antigen 125; CA 19-9, cancer antigen 19-9; CEA, carcinoembryonic antigen; CPK, creatine phosphokinase; GGT, gamma-glutamyltransferase

Non-cumulative and reversible neutropenia and transient gamma-glutamyltransferase (GGT) increase were the most common laboratory abnormalities seen with trabectedin plus PLD as compared to baseline values (Fig. 2). In contrast, other hematological, biochemical and tumor marker parameters seen during the study stayed similar to the baseline values. Overall, 44 (57.1%) of patients had at least one of 278 trabectedin-related AEs of any grade. Of those, 197 AEs (70.9%) have totally recovered and 70 AEs (25.2%) were considered to be tumor-related. Most common grade 3/4 trabectedin-related AEs were leukopenia (*n* = 14, 18.2% of patients), neutropenia (*n* = 12, 15.6%), thrombocytopenia (*n* = 7, 9.1%), alanine aminotransferase (ALT; *n* = 6, 7.8%) increase, aspartate aminotransferase (AST; *n* = 5, 6.5%) increase, and nausea/vomiting (*n* = 4, 5.2% each) (Table 4). Moreover, 49 (63.6%) of patients experienced at least one of 263 PLD-related AEs of any grade. Most of PLD-related AEs have totally recovered (198 AEs, 75.3%), whereas 76 AEs (28.9%) were considered to be tumor-related. Neutropenia (*n* = 14, 18.2%), leukopenia (*n* = 12, 15.6%), thrombocytopenia (*n* = 8, 10.4%), and nausea/vomiting (*n* = 4, 5.2% each) were the most common grade 3/4 PLD-related AEs (Table 4). No deaths attributed to drug-related AEs or unexpected AEs occurred.

**Table 4:** Treatment-related hematological and non-hematological toxicities

Treatment-related AEs NCI-CTC grade (≥ 10% of patients)	Grade 1		Grade 2		Grade 3		Grade 4		Total	
	*n*	%	*n*	%	*n*	%	*n*	%	*n*	%
Trabectedin-related AEs										
Nausea	5	6.5	13	16.9	4	5.2			22	28.6
Vomiting	3	3.9	4	5.2	4	5.2			11	14.3
ALT increased	2	2.6			5	6.50	1	1.3	8	10.4
AST increased	2	2.6	2	2.6	4	5.2	1	1.3	9	11.7
Neutropenia	1	1.3	2	2.6	8	10.4	4	5.2	15	19.5
Thrombocytopenia	2	2.6	3	3.9	2	2.6	5	6.5	12	15.6
Leukopenia	1	1.3	5	6.5	10	13.0	4	5.2	20	26.0
PLD-related AEs										
Nausea	5	6.5	13	16.9	4	5.2			22	28.6
Vomiting	3	3.9	5	6.50	4	5.2			12	15.6
Neutropenia	1	1.3	2	2.6	8	10.4	6	7.8	17	22.1
Thrombocytopenia	2	2.6	3	3.9	2	2.6	6	7.8	13	16.9
Leukopenia	2	2.6	6	7.8	8	10.4	4	5.2	20	26.0

Data shown are numbers and percentage of patients with available data

*AE* adverse event, *ALT* alanine aminotransferase, *AST* aspartate aminotransferase, *NCI-CTC* National Cancer Institute Common Toxicity Criteria

Sixty-two AEs regardless of causality with the study drug that occurred in 40 patients (52.0%) were considered as serious adverse events (SAEs). The most frequent SAEs were ileus (*n* = 7, 9.1%) followed by abdominal pain and vomiting (*n* = 4, 5.2% each). Overall, AEs were classified as serious either because required prolonged in-patient hospitalization (*n* = 59, 95.2%), were considered life-threatening (*n* = 7 SAEs, 11.2%), resulted in non-treatment related death (*n* = 6, 9.7%), were deemed as important medical events (*n* = 2, 3.2%) or had missing causality (*n* = 1, 1.6%). Finally, 53 of all SAEs (85.5%) have totally recovered, with no need for treatment changes or other action in more than 60% of SAEs.

### Subsequent therapies

The median time to next treatment (TTNT), counted from the last trabectedin plus PLD dose to next treatment switch, was 4.31 month (CI 95% 1.8–5.7). As recorded during the follow-up visits at 6 and 12 months, 42 out of 52 presented patients (80.8%) at the first and 15 of 20 evaluated patients (75%) at the second follow-up visit received a subsequent anticancer therapy. Of those, most patients received chemotherapy: 39 out of 42 patients at 6 months (92.9%) and 12 of 15 patients (80.0%) at 12 months after the end of the study. An analysis of given chemotherapies evidenced that at both follow-up visits the number of patients who received non-platinum drugs (at 6 months *n* = 25, 64.1%; at 12 months *n* = 8, 66.7%) as first subsequent chemotherapy doubled those who received platinum-based chemotherapy (at 6 months *n* = 14, 35.9%; at 12 months *n* = 4, 33.3%). The best responses following the subsequent treatment/s have not been documented.

## Discussion

The OVA-YOND study is the first ever non-interventional study that prospectively evaluated the outcomes of the non-platinum combination of trabectedin plus PLD in routine clinical practice in women with a platinum-sensitive relapse of ovarian cancer. Although randomized controlled clinical trials are the golden standard of medical evidence, their applicability to daily clinical practice and generalizability to heterogeneous and diverse patient populations should be verified in non-interventional studies to gain the real-life validity. The present unselected patients represented a pretreated and heterogeneous population with multiple comorbidities and with high grade serous carcinoma (FIGO stage IIIC–IV) in ~ 70% of patients, which truly reflects a real-life situation. Moreover, the fact that a complete primary resection was achieved in 26% of patients only and that 23.4% of patients underwent a recurrence surgery, of which 5.2% achieved a complete resection, is strongly related with a poor prognosis of therapy and largely represents the outcomes from non-specialized centers. Therefore, the present results should be considered representative of patient demographics, clinical practice and outcomes in the real-life practice across Germany. On the other hand, in spite of the fact that the eligibility criteria of this study were less restrictive than those of prospective clinical trials, the patients were treated according the terms of the marketing authorization.

In our study, the analyzed patient population was older (median age 66 vs. 56 years), in worse condition with performance status 0/1 in 80.5 vs. 97.3% of patients and more pretreated than that included in the pivotal randomized phase III OVA-301 study, where the inclusion criteria allowed only one previous line of treatment (Monk et al. 2010). In contrast, the baseline characteristics of patients of this observational study are similar to those reported in other retrospective analyses, in which the patients were pretreated with a median of three chemotherapy lines (Moriceau et al. 2016; Nicoletto et al. 2015) (Table 5). In the present study, trabectedin plus PLD administration resulted in an ORR of 31.2% and a median PFS and OS of 6.3 and 16.4 months, respectively. This benefit was observed regardless of the method of response assessment or disease histology, previous lines of systemic therapy, or any other clinical considerations (e.g., age or line of treatment). Recognizing that comparisons cannot be established, and merely with the aim of putting the findings yielded here in wider context, the benefit in PFS and OS in the OVA-YOND study was similar to those observed in two real-world retrospective analyses, which reported a median PFS of 6.1 and 6.7 months and a median OS of 16.3 and 17.6 months, respectively (Moriceau et al. 2016; Nicoletto et al. 2015). In the pivotal OVA-301 trial, data obtained from the platinum-sensitive subset of patients showed an ORR of 35.3% with a median PFS and OS of 9.2 and 27.0 months. While the ORR obtained in the present study is comparable to that of OVA-301, the differences in PFS and OS with the pivotal trial may be partially explained by the fact that the patients in OVA-301 study were considerably more selected, younger and less pretreated, and by the immaturity of the data in the present study (23.4 and 46.8% of patients were censored for PFS and OS, respectively). However, the overall data in real-life patients seem to be consistent with those observed in the OVA-301 study and support that trabectedin plus PLD maintains antitumor activity regardless of the number of previously administered chemotherapy lines. The benefit of trabectedin plus PLD was also supported by the fact that subsequent anticancer therapy was given after a median time of 4.31 months, demonstrating the activity of the combination in real-life settings. Moreover, the facts that in most patients (~ 65%) a non-platinum-based regimen was the first choice of subsequent chemotherapy after trabectedin plus PLD additionally confirms that non-platinum drugs are largely recommended as the preferred palliative option for heavily pre-treated patients with ROC.

**Table 5:** Relevance of OVA-YOND results within the context of trabectedin plus pegylated liposomal doxorubicin treatment of recurrent platinum-sensitive ovarian cancer

Studies	*n*	Age Median (range)	Prior chemotherapy lines Median (range)	ECOG PS score 0/1	Platinum-sensitive patients (PFI ≥ 6 months)		
					ORR (%)	Median PFS (months)	Median OS (months)
Real-world outcomes							
Moriceau et al. (2016)^a^	17	61 (48–78)	3 (1–9)	94.1	53.0	6.7	17.6
Nicoletto et al. (2015)	34	60 (26–79)	3 (2–10)	NR	32.4	6.1	16.3
OVA-YOND study							
Runnebaum et al.	77	66 (40–78)	2.0 (1–6)	80.5	31.2	6.3	16.4

*ECOG* Eastern Cooperative Oncology Group, *NR* not reported, *ORR* overall response rate, *OS* overall survival, *PFI* platinum-free interval, *PFS* progression-free survival, *PS* performance status

^a^Ten patients (59%) had platinum-resistant disease with a PFI < 6 months

While the prognosis of primary therapy is closely related to the disease stage at diagnosis and the extent of residual disease following surgery, the PFI and performance status are two of the most important prognostic factors in ROC. Unfortunately, there is limited published literature on the evaluation of cancer-related symptoms in women with recurrent platinum-sensitive ovarian carcinoma. In our study, more than a half of the patients reported either gain or not changed bodyweight throughout the study, whereas the number of patients with a good performance status score of 0/1 decreased from 80.5% at baseline to 50.6% at the therapy end. No significant changes were observed in heart rate, blood pressure and electrocardiogram during the study. These data indicate a low symptomatic worsening under trabectedin plus PLD treatment, which was basically caused by the natural course of disease. It is also important to notice that the proportion of patients treated on an outpatient setting was 88.3%, suggesting a good performance status in most of them.

In accordance with the non-interventional setting, this type of study carries some weaknesses such as the exact time points and method of response assessment were not previously fixed but were done according to the clinician’s discretion and usual clinical practice, the number of enrolled patients was low, the patients were less frequently monitored than in clinical trials, the response evaluation did not distinguish between the platinum-sensitive subgroups and the study’s findings were neither centrally reviewed nor confirmed. Therefore, our results must be interpreted with caution as they may be subject to bias.

Although overlapping toxicities between trabectedin and PLD have surely occurred to some degree, the safety and tolerability of trabectedin plus PLD were consistent with extensive prior experience and reports reflecting the well-characterized transient and non-cumulative toxicities of bone marrow suppression and hepatotoxicity (Le Cesne et al. 2012; Monk et al. 2010). According with the terms of the marketing authorization, there are no pre-defined limits to the number of cycles administered as it is indicated to continue the treatment while clinical benefit is noted. Herein, the median number of cycles received per patient was the same as that reported in the pivotal OVA-301 trial (median 6 cycles, range 1–21) (Monk et al. 2010). Moreover, more than 50% of patients received ≥ 6 cycles, suggesting an acceptable safety profile that allowed prolonged treatment. No drug-related deaths, new or unexpected AEs or qualitative differences in the AEs were observed.

In conclusion, the findings of this non-interventional, prospective real-life study consistently support that trabectedin plus PLD confers clinically meaningful long-term benefits to pre-treated patients with platinum-sensitive ROC, associated with poor prognosis, being consistent with the findings reported in the prior phase III study. Large randomized trials are warranted to show that platinum-free combinations in platinum-sensitive disease may effectively allow patients’ recovery from previous platinum-associated adverse effects with similar or improved oncological outcome.
